# Achieving a polio free world

**DOI:** 10.1186/s12916-014-0116-3

**Published:** 2014-07-03

**Authors:** Mohammad Ali, David A Sack

**Affiliations:** 1 Department of International Health, Johns Hopkins Bloomberg School of Public Health, 615 N.Wolfe Street / E5543, Baltimore 21205, Maryland, USA

**Keywords:** Polio, Spatial model, Risk area, OPV

## Abstract

Recently, the World Health Organization (WHO) declared that the spread of polio is an international public health emergency, and a coordinated international response is sought. Although the importance of such a response is recognized, there are challenges to stopping the spread of polio and achieving a polio free world. The most important issue is directing limited national resources to the specific areas where polio is endemic. In an article published in *BMC Medicine*, Upfill-Brown and his colleagues recognized this problem and successfully identified the potential risk areas in Nigeria using a validated spatial predictive model of wild poliovirus circulation. They also showed that a lower vaccine-derived population immunity is associated with the probability of a higher number of wild poliovirus cases within a district. Identification of the potential risk areas and understanding the magnitude of risk may help direct limited resources of the endemic countries to areas most at risk to maximize the impact of interventions and motivate the people to participate in the intervention program. These efforts are crucial if these endemic countries hope to eradicate polio.

Please see related research article: http://www.biomedcentral.com/1741-7015/12/92.

## Background

Polio was first traced as a disease of a separate clinical entity by Michael Underwood in 1789 [[1]]. The disease mainly affects children under five years old. Three types of poliovirus, designated as 1, 2 and 3, may cause polio [[2]], although only type 1 appears to be circulating currently. Type 2 was last reported in 1999 and type 3 was last reported in 2012. Humans are the only reservoir of poliovirus [[3]]. The virus is transmitted person-to-person through fecal-oral and oral-oral routes, or less frequently by water and milk [[4],[5]]. It multiplies in the intestine and spreads to the central nervous system, causing paralysis in 1 in every 200 infections [[6]], with a case fatality of 5% to 10% [[7]]. Polio became a major public health issue in late Victorian times when major epidemics occurred in the United States and Europe.

Significant progress toward polio eradication has been achieved during 2012/2013 and, as a result, 80% of the world’s population now live in WHO regions certified as polio-free [[8]]. However, the poliovirus remains endemic in three countries: Pakistan, Afghanistan and Nigeria. During 2014, as of May 20, wild poliovirus 1 has already spread to central Asia, the Middle East (Syria to Iraq) and to Central Africa (Cameroon to Equatorial Guinea) [[8]]. Attacks on vaccination workers in Pakistan are thought to have allowed the virus to spread across borders. Infections in Pakistan, Cameroon and Syria increase the risk for further wild poliovirus exportations in 2014. Accordingly, WHO declared the spread of polio to be an international public health emergency [[9]]. This is only the second time in the WHO's history that it has made such a declaration, the first being during the swine flu pandemic of 2009. The WHO also listed Afghanistan, Equatorial Guinea, Ethiopia, Iraq, Israel, Somalia and Nigeria as posing an ongoing risk for new wild poliovirus exportations in 2014.

Although the importance of a coordinated international response is recognized, there are challenges to stopping the spread of polio and achieving a polio-free world. One important issue is how to direct the limited resources within the polio endemic countries. Although all children need to receive vaccine, decisions are still needed to know where special efforts are required. To assist with these decisions, Upfill-Brown and colleagues identified the potential risk areas in Nigeria using a spatial modelling technique and the Nigerian Acute Flaccid Paralysis surveillance database maintained by the Nigerian WHO [[10]]. Their results showed that lower vaccine-derived population immunity is associated with the probability of a higher number of wild poliovirus cases within a district. Based on the results of their spatial model, they also estimated that under-five oral polio vaccine (OPV)-induced population immunity would decrease by 8% to 9% in a six month period in the absence of any vaccination. Although a substantial amount of residual spatial variation remains in their model due to the paucity of the data on poverty, malnutrition, sanitation and level of health services, all of which influence wild polio virus transmission potential and population vaccine efficacy [[11]], the predictive accuracy of their model was found to be very high (more than 85%). Such model results are useful for political and religious advocacy as well as public health policy makers to allocate resources to the highest risk areas.

### The challenges to eradicating polio from the globe

The global program’s focus is on stopping polio transmission in the remaining three endemic countries. Transmission of wild poliovirus has never been eliminated in Nigeria, and neighboring countries have become re-infected by strains originating from Nigeria [[12]]. Presently, Nigeria accounts for almost 60% of world’s polio cases; thus, this country is considered to be the biggest threat to a polio-free world [[13]]. Besides Nigeria, wild polioviruses continue to persist in Pakistan and one part of Afghanistan. Eight other countries are currently responding to outbreaks related to imported viruses [[14]]. To stop the transmission of poliovirus, every child in the three endemic countries needs to be immunized, but security risks limit access in some of the endemic areas of Afghanistan and Pakistan. By inhibiting vaccination programs, these security risks pose a continuing threat to achieving a polio-free world. However, as mentioned by Upfill-Brown *et al*., the impact of the vaccination efforts will be maximized if one can efficiently direct limited resources and specially trained personnel, along with community outreach activities and satellite vaccinator tracking to the most at-risk areas. A validated predictive model of wild poliovirus circulation would, therefore, greatly inform prioritization efforts by accurately forecasting areas at greatest risk, thus enabling the greatest effect of the program.

The threat of the spread of polio between countries, especially between these endemic countries and their neighbors's risks the health and wellbeing of the countries of the region and also increases the costs of its containment because of the need for supplementary immunization campaigns. However, just as polio spreads between countries, it also spreads within these endemic countries and immunizing these areas which are the source of the spread should reduce the overall disease burden within these endemic countries and should facilitate the elimination of the virus in these endemic countries.

While vaccination is crucial, it must also be realized that OPV is less effective in children in many developing countries. The reasons for the lower effectiveness of an oral vaccine are not known, but some potential factors may include tropical enteropathy, malnutrition, interference from maternal breast milk or serum antibodies, alterations in the gut microbiota, and genetic susceptibility [[15]]. Other reasons could include (in some cases) lack of a cold chain resulting in damage to the live virus vaccine; interference by infection with endogenous enteroviruses [[16]]; or interference via co-administration of OPV and rotavirus vaccines [[17]].

## Conclusions

It is important that Nigeria become polio free to establish a polio-free Africa. Similarly, Pakistan and Afghanistan need to be polio free to establish a polio-free Asia. The predictive model of UpFill-Brown *et al*. demonstrated a clear link between vaccination coverage and the magnitude of the risk of poliomyelitis in Nigeria.

The funding constraints as well as the logistic challenges of OPV campaigns are huge. One must attempt to reach more than 90% of young children through mass OPV immunization campaigns in countries with reestablished transmission; this task is similar in scale to those tasks in the remaining endemic countries where conflict, insecurity and weak public services complicate eradication operations [[14],[18]]. These issues need to be successfully addressed to achieve a polio free world.

## Competing interests

The authors declare that they have no competing interests.

## Authors’ contributions

Both authors contributed to conception of the article. MA made the first draft of the article. Both authors were involved in editing and revision of the manuscript and both read and approved the final manuscript.
